# A80G polymorphism of reduced folate carrier 1 (*RFC1*) and C776G polymorphism of transcobalamin 2 (*TC2*) genes in Down's syndrome etiology

**DOI:** 10.1590/S1516-31802008000600007

**Published:** 2008-11-06

**Authors:** Joice Matos Biselli, Daniela Brumati, Vivian Fernanda Frigeri, Bruna Lancia Zampieri, Eny Maria Goloni-Bertollo, Érika Cristina Pavarino-Bertelli

**Keywords:** Down syndrome, Polymorphism, genetic, Folic acid, Nondisjunction, genetic, Transcobalamins, Síndrome de Down, Polimorfismo genético, Ácido fólico, Não-disjunção genética, Transcobalaminas

## Abstract

**CONTEXT AND OBJECTIVE:**

There is evidence that polymorphisms of genes involved in folate metabolism may be associated with higher risk that mothers may bear a Down's syndrome (DS) child. This study therefore had the objective of investigating the A80G polymorphism of the reduced folate carrier 1 (*RFC1*) gene and the C776G polymorphism of the transcobalamin 2 (*TC2*) gene as maternal risk factors for DS among Brazilian women.

**DESIGN AND SETTING:**

Analytical cross-sectional study with control group, at Faculdade de Medicina de São José do Rio Preto (Famerp).

**METHODS:**

Sixty-seven mothers of DS individuals with free trisomy 21, and 113 control mothers, were studied. Molecular analysis of the polymorphisms was performed by means of the polymerase chain reaction with restriction fragment length polymorphism (PCR-RFLP), followed by electrophoresis on 2% agarose gel.

**RESULTS:**

The frequencies of the polymorphic alleles were 0.51 and 0.52 for *RFC1* 80G, and 0.34 and 0.34 for *TC2* 776G, in the case and control groups, respectively. Thus, there were no differences between the groups in relation to either the allele or the genotype frequency, for both polymorphisms (P = 0.696 for *RFC1* A80G; P = 0.166 for *TC2* C776G; P = 0.268 for combined genotypes).

**CONCLUSION:**

There was no evidence of any association between the *RFC1* A80G and *TC2* C776G polymorphisms and the maternal risk of DS in the sample evaluated.

## RESUMO

**CONTEXTO E OBJETIVO:**

Considerando as evidências de que polimorfismos em genes envolvidos no metabolismo do folato podem estar associados ao risco materno elevado para a síndrome de Down (SD), o objetivo deste estudo foi investigar os polimorfismos A80G do gene carregador de folato reduzido^1^ (*RFC1*) e C776G do gene transcobalamina^2^ (*TC2*) como fatores de risco maternos para a SD em mulheres brasileiras.

**TIPO E ESTUDO LOCAL:**

Estudo transversal analítico com grupo controle, realizado na Faculdade de Medicina de São José do Rio Preto (Famerp).

**MÉTODOS:**

Foram avaliadas 67 mães de indivíduos com trissomia livre do 21 e 113 mães de indivíduos sem a síndrome. A análise molecular dos polimorfismos foi realizada pela técnica de reação em cadeia da polimerase/polimorfismo de comprimento fragmentos de restrição (PCR-RFLP), seguida por eletroforese em gel de agarose 2%.

**RESULTADOS:**

As freqüências dos alelos polimórficos foram de 0,51 e 0,52 para *RFC1* 80G e 0,34 e 0,34 para *TC2* 776G nos grupos caso e controle, respectivamente. Assim, não houve diferença nas freqüências alélicas e genotípicas para ambos os polimorfismos entre os grupos (P = 0,696 para *RFC1* A80G; P = 0,166 para *TC2* C776G; p = 0,268 para genótipos combinados).

**CONCLUSÃO:**

Não há evidência de associação entre os polimorfismos *RFC1* A80G e *TC2* C776G e o risco materno para a SD na casuística avaliada.

## INTRODUCTION

Down's syndrome (DS) or trisomy 21 is a genetic disease associated with abnormal chromosomal segregation.^1^ Free trisomy 21 is found in 95% of DS cases and is due to chromosome 21 nondisjunction, which in most cases occurred during maternal meiosis.^2^

The risk factors for meiotic nondisjunction are not very clear, except for advanced maternal age.^3,4^ Folic acid has an important role in the process of genetic material distribution during cell division, because of its importance to cellular methylation reactions.^5^ The folate metabolism is responsible for synthesizing S-adenosylmethionine (SAM), the main donor of methyl groups for cellular methylation reactions.^6^ It has been shown that DNA methylation is important for maintaining centromeric chromatin stability and plays an important role in chromosomal segregation.^7,8^

Polymorphisms of genes that encode enzymes involved in folate metabolism have been associated with the etiology of DS.^9-12^ A polymorphism of the reduced folate carrier 1 (*RFC1*) gene consisting of an adenine-to-guanine substitution at position 80 (A80G) has been associated with altered concentrations of products derived from the folate metabolic pathway.^13,14^ This has been indicated as a maternal risk factor for DS, in combination with other polymorphisms involved in this metabolism.^9,11^ The *RFC1* gene encodes the reduced folate carrier 1 protein, which plays a role in folic acid absorption, thereby transporting 5-methyltetrahydrofolate, the metabolically active form of folate, into a variety of cells.^15^

Polymorphisms of genes encoding cobalamin-transporting proteins such as transcobalamin 2 (*TC2*) may interfere with the availability of this vitamin in the organism.^16,17^ Cobalamin (vitamin B_12_) plays an important role in folate metabolism because of its action as cofactor for methionine synthase (MTR) enzyme.^18^ Thus, genetic variants in the *TC2* gene possibly have an influence on cellular methylation reactions and on the risk that the mother may bear a DS child. The continuing studies on the etiology of DS are motivated by the importance of this subject for families, given the repercussions from the birth of a child with DS.

## OBJECTIVE

This study had the objective of investigating the *RFC1* A80G and *TC2* C776G polymorphisms as maternal risk factors for DS.

## METHODS

This was an analytical cross-sectional study with a control group carried out at Faculdade de Medicina de São José do Rio Preto (Famerp). After informed consent had been obtained, peripheral blood samples were taken from 67 mothers of DS individuals with free trisomy 21 (case group) and 113 mothers of individuals without the syndrome (control group). Mothers of DS individuals with translocation or mosaicism were not included in the study. The average age among the mothers at the time of blood collection was 36.7 ± 10.4 years in the case group and 40.5 ± 8.2 years in the control group.

For molecular analysis, DNA was extracted from peripheral blood leukocytes, as described by Miller et al.^19^ The *RFC1* A80G polymorphism was investigated by means of the polymerase chain reaction with restriction fragment length polymorphism (PCR-RFLP), using the primer sequences described by Födinger et al.^20^ and the enzyme *CfoI* to recognize the polymorphic site. The evaluation of the *TC2* C776G polymorphism was performed by means of PCR-RFLP using the forward primer 5’- CAT CAG AAC AGT GCG AGA GG –3’ and the reverse primer 5’- GTG CCA GAC AGT CTG GGA AG –3’, and the enzyme *ScrF1* to recognize the polymorphic site.^21^ The resulting fragments from enzyme digestion were then subjected to electrophoresis on 2% agarose gel.

The chi-squared test was used for statistical analysis on genotype frequencies. Comparisons of maternal age between the groups was carried out by using Student's t test; P values ≤ 0.05 were taken to be statistically significant.

## RESULTS

Table 1 presents the distribution of the groups according to maternal age. The mean maternal age in the case group was 32 ± 8.6 years, and in the control group, it was 27.4 ± 5.5 years (P < 0.0001).

**Table 1:** Distribution of the case (n = 67) and control (n = 113) groups according to maternal age

Maternal age	Case n (%)	Control n (%)
≤ 20 years	6 (9.0)	15 (13.3)
> 20 – 30 years	26 (38.8)	66 (58.4)
> 30 – 35 years	7 (10.4)	23 (20.3)
> 35 years	28 (41.8)	9 (8.0)

The frequencies of the polymorphic alleles *RFC1* 80G and *TC2* 776G were 0.51 and 0.34 in the case group and 0.52 and 0.34 in the control group, respectively. The genotype distribution of the polymorphisms is presented in Table 2. There were no significant differences in genotype distribution between the groups (P = 0.696 for *RFC1* A80G; P = 0.166 for *TC2* C776G).

**Table 2:** Genotype distribution of the *RFC1* A80G and *TC2* C776G polymorphisms between the case (n = 67) and control (n = 113) groups

Genotypes	Case n (%)	Control n (%)
*RFC1* A80G		
AA	14 (20.9)	30 (26.5)
AG	33 (49.3)	49 (43.4)
GG	20 (29.9)	34 (30.1)
*TC2* C776G		
CC	32 (47.8)	48 (42.5)
CG	24 (35.8)	54 (47.8)
GG	11 (16.4)	11 (9.7)

Analysis of the combined genotypes of the two polymorphisms did not show any statistically significant differences between the groups (P = 0.268). Nor did the genotypes differentiate between the groups when only women with maternal age under 35 years were considered (P = 0.714 for *RFC1* A80G; P = 0.166 for *TC2* C776G; P = 0.759 for combined genotypes).

## DISCUSSION

Several technological advances in molecular cytogenetics, making it possible to identify most chromosomal aberrations (both structural and numerical) and the genes responsible for some diseases, have been achieved.^22^ Nevertheless, although the chromosomal basis of DS has been well characterized, the etiology of chromosome 21 nondisjunction remains unclear.

Among the risk factors associated with DS occurrence, advanced maternal age is the best established factor.^3,4^ In fact, it has been shown that the frequency of chromosomal aberrations becomes greater with advancing maternal age,^3,24,25^ especially with regard to trisomy of chromosomes 13, 18 and 21 in women who give birth when over 35 years old.^26^ According to data from the Latin American Collaborative Study of Congenital Malformations (ECLAMC), 40% of live births with DS were born from mothers who were between 40 and 44 years old, although women of this age account for only 2% of all births.^27^ In our study, the mean maternal age was significantly higher in the case group than in the control group. This corroborates with data in the literature regarding the association between maternal age and the risk of DS.

Occurrences of births of children with DS among young mothers suggests that advanced maternal age is not the only risk factor involved. Investigation of genetic polymorphisms that lead to abnormalities in folate metabolism products is the approach furthest investigated at the present time.^28^

James et al.^29^ reported that mothers with DS children have abnormal folate metabolism. They suggested that a variant of the methylenetetrahydrofolate reductase gene (*MTHFR* C677T), which regulates cellular methylation reactions, could lead to DNA hypomethylation and consequently to chromosomal segregation errors. Other genes involved in the folate metabolic pathway have been investigated as maternal risk factors for DS, such as *RFC1, MTR*, methionine synthase reductase (*MTRR*), cystathionine B-synthase (*CBS*). Evidence indicating the contribution of variants of these genes towards the maternal risk of bearing a DS child has been put forward.^9-12^

However, the few studies that have evaluated the influence of *RFC1* A80G polymorphism, including a previous study by our group,^9^ did not observe any association between this variant *per se* and the maternal risk of bearing a DS child,^9,10,30^ thereby corroborating the present study. Higher maternal risk of DS has been observed in the presence of this polymorphism, in combination with other genetic polymorphisms relating to folate metabolism, such as *MTHFR* C677T, *MTHFR* A1298C and *MTR* A2756G.^9,10^ This may be due to the small impact of *RFC1* A80G polymorphism on the affinity and transport efficiency of the variant enzyme in relation to the wild-type enzyme.^31^

With regard to *TC2* C776G polymorphism, the present study is, to our knowledge, the first to investigate the contribution of polymorphisms of the cobalamin-transporting gene (an important cofactor for folate metabolism), in relation to the maternal risk of bearing a DS child. There is evidence for an association between this genetic variant and the maternal risk of bearing a child with neural tube defects,^32^ which are influenced by genetic determinants involved in folate metabolism.

Evidence showing a higher frequency of DS cases in families with a risk of neural tube defects, and vice versa,^33^ strengthens the notion that the same genetic determinants of folate metabolism influence both disorders.^34^ However, in the present study, no association was observed between the *TC2* C776G polymorphism and the maternal risk of bearing a DS child. It is widely accepted that supplementation or fortification with folic acid reduces the risk of neural tube defects,^35,36^ and it is believed that supplementation with cobalamin in combination with folate could contribute towards reducing this risk.^18,32^

## CONCLUSION

No evidence for an association between *RFC1* A80G and *TC2* C776G polymorphisms and the maternal risk of bearing a DS child was observed in this study. Thus, further studies including these and other polymorphisms involved in folate metabolism could provide a better understanding of the role of genetic variants in the etiology of the chromosomal nondisjunction that results in DS.
